# Investing in Exposure

**DOI:** 10.1371/journal.pmed.0030278

**Published:** 2006-06-27

**Authors:** Abdul Moiz Khan

**Affiliations:** **1**Jhang, PunjabPakistan

The problems of the overwhelming amount of medical literature have been thoughtfully highlighted in the *PLoS Medicine* editorial “Drowning or Thirsting: The Extremes of Availability of Medical Information” [ 1]. It is of vital importance to medical professionals that they improve on their skills of sifting through the huge amounts of literature that are made available to them every month. But this task becomes exponentially more difficult in the less-developed world, where basic knowledge of medical literature is limited [ 2].

There are no alternatives to the good analytical skills that come through continued exposure to medical literature. This exposure should begin at the medical student level. Exercises such as those requiring medical students to analytically criticize medical literature can go a long way in developing reading skills.

The second issue is that of disseminating the literature that has been published. Access to reliable health information has been described as “the single most cost-effective and achievable strategy for sustainable improvement in health care” [ 3].

A useful strategy could be making less-expensive paper versions of medical journals widely available in less-developed countries. Publishers should be willing to look into this approach. Regional copies of journals can be produced locally and inexpensively. This will boost the circulation of medical journals. Medical professionals will not mind a little compromise in the quality of paper in a journal, so long as they are able to afford it at a low price.

The business model of offshore call centers can serve as a useful one in the case of publishing low-cost copies of medical journals. Companies have shifted their call centers to less-developed countries where services are available at very low costs. The costs of publishing are likewise bound to be cheaper in the less-developed countries; therefore, journals can be produced at affordable prices. If this model can be followed by multinationals, why is it not possible for a cause as noble as publishing medical literature?

Another approach could be that journal volumes could be condensed, so that only research relevant to the local area is published in the local version of the journal.

The Internet's widely spreading use as a resource can be of vital value in substantially quenching the thirst of professionals. The wide availability of Internet access in Pakistan [ 4], for example, helps the cause of disseminating information. Internet access such as that given through the platform of HINARI or the Ptolemy project [ 5] is also a viable option. But these networks need to be expanded to include more countries and individuals [ 6].

One should hope that investing in improving exposure of health professionals to medical literature will help improve their practices and the quality of healthcare that they provide to impoverished populations in their local area. The need of the hour is to be innovative and be ready to embrace new and thoughtful ideas for the collective good of humanity.
